# Law Enforcement Officer Knowledge of, Attitudes Toward, and Willingness to Use Extreme Risk Protection Orders

**DOI:** 10.1001/jamanetworkopen.2023.38455

**Published:** 2023-10-19

**Authors:** Veronica A. Pear, Alaina De Biasi, Amanda Charbonneau

**Affiliations:** 1 Violence Prevention Research Program, Department of Emergency Medicine, University of California, Davis, Davis; 2 Department of Criminology & Criminal Justice, Wayne State University, Detroit, Michigan; 3 RAND Corporation, Santa Monica, California

## Abstract

**Importance:**

Understanding knowledge of, attitudes toward, and willingness to use extreme risk protection order (ERPO) laws among law enforcement officers (LEOs) can inform efforts to improve implementation of this underused firearm violence prevention strategy.

**Objective:**

To characterize LEOs’ knowledge of, attitudes toward, and willingness to use ERPOs across a range of scenarios.

**Design, Setting, and Participants:**

A cross-sectional online survey, fielded from April 5 to August 30, 2021, was conducted in all 19 states and the District of Columbia with an ERPO law in 2021. A nonprobability sample of active-duty LEOs was used.

**Exposure:**

Being a LEO in a state with an ERPO law.

**Main Outcomes and Measures:**

Survey participants answered questions about their familiarity with and opinions on ERPO laws, as well as whether they would agree with using an ERPO in a variety of specific case scenarios. The analysis included an exploration of whether within-scenario differences, such as ERPO respondent race or gender, affected agreement by randomly assigning survey participants to 1 of 2 versions of each scenario.

**Results:**

A total of 600 eligible individuals started the survey, and 283 survey participants were included in the analysis. The analytic sample consisted mostly of cisgender men (85.2%) and non-Hispanic White (71.4%) LEOs. Participants represented 14 states and the District of Columbia, with 53.7% living in California. Most participants (81.3%) were very or somewhat familiar with ERPO laws and 56.2% had received ERPO training. Opinions about ERPO laws were generally favorable but varied by self-identified political ideology. Across all scenarios, most participants supported using an ERPO; however, support was highest in cases involving intimate partner violence (71.4%-78.6%) and lowest in cases involving suicidality (54.2%-73.3%). Across all scenarios, LEOs with ERPO training or experience were substantially more likely to agree with using ERPOs than those without. None of the randomly assigned within-scenario differences were associated with differences in ERPO support.

**Conclusions and Relevance:**

In this survey study of LEOs in states with ERPO laws, many officers had not received training on their use. Additionally, while conservative political ideology was associated with less favorable views of ERPOs, training and experience with ERPOs was associated with greater support for their use across a range of scenarios. These findings suggest that LEO training on ERPOs may promote their uptake and improve implementation.

## Key Points

**Question:**

How familiar are law enforcement officers (LEOs) with extreme risk protection orders (ERPOs), and in what circumstances do they consider ERPOs to be appropriate?

**Findings:**

This survey study of 283 active-duty LEOs in states with ERPO laws found that knowledge of ERPOs was high, but willingness to use ERPOs varied. Across all provided scenarios, LEOs with ERPO training or experience were substantially more likely to support the use of ERPOs than those without.

**Meaning:**

The findings of this study suggest that training LEOs on ERPO laws may improve their implementation.

## Teaser

This survey study examines the understanding of and opinions on the use of extreme risk protection order laws in active-duty law enforcement officers.

## Introduction

Extreme risk protection order (ERPO) laws temporarily prohibit individuals judged to be at high risk of harming themselves or others from purchasing or possessing firearms. Currently, 21 states and the District of Columbia have ERPO laws, with most enacted after the school shooting in Parkland, Florida, in February 2018. The ERPO laws seem to be effective at preventing firearm suicide^1,2,3^ and possibly mass shootings,^4,5,6^ but slow uptake has limited their impact.^7,8^

Implementation and use of ERPOs largely depends on law enforcement officers (LEOs), who are permissible petitioners (ie, the individuals requesting ERPOs) in all ERPO states but Vermont and are the only permissible petitioner in 4 states.^9^ Even in states that allow other petitioner types, LEOs file most petitions.^8,10,11,12^ However, lack of awareness, limited training, and local politics can all serve as barriers to officers using ERPOs.^13^ Prior surveys suggest that knowledge of these laws is limited among the general population and certain petitioners, with approximately two-thirds of California adults and more than 70% of Maryland physicians (who are permitted petitioners in that state) at the Johns Hopkins Hospital in Baltimore never having heard of the law.^14,15^ Training for LEOs on ERPOs might help raise awareness and provide guidance for implementation, but little is known about how common such training is.

However, even if LEOs are aware of and trained to use their state’s ERPO law, implementation may be stymied by local or personal politics. A number of sheriffs, most notably in Colorado, declared their counties “Second Amendment Sanctuaries,” indicating a refusal to petition for ERPOs (although approximately 25% of these counties issued ERPOs in Colorado within the law’s first year^12^). While most people support ERPOs after learning about the law,^15^ including firearm owners,^16^ key informant interviews previously suggested that officers are likely to have personal political reservations about using ERPOs.^13^ To the extent that politics are preventing individuals from having meaningful access to ERPOs, there is inequity in the law’s implementation (ie, if local law enforcement refuses to petition for or serve an ERPO on political grounds, individuals living within their jurisdiction are faced with a substantial barrier to using ERPOs that others do not face).

Because LEOs play an essential role in the implementation of ERPO laws, we sought to learn more about officer knowledge of, attitudes toward, and willingness to use this law. Prior key informant interviews exploring ERPO implementation have included LEOs, but these samples are small and typically include officers from limited geographic locations.^2,3,13^ Building on this earlier work, this study presents results from a survey of LEOs in the District of Columbia and all 19 states with an ERPO law in 2021 that explored officer knowledge, training, and opinions about ERPO laws as well as willingness to use ERPOs across a range of scenarios. To our knowledge, this is the first study of its kind. Findings may directly inform LEO implementation of ERPO laws and may be of interest to those working in law enforcement, policy, and violence prevention.

## Methods

### Design

This was a cross-sectional online survey of LEOs in all ERPO states and the District of Columbia, designed by 2 of us (V.A.P. and A.C.) and administered by Qualtrics from April 5 to August 30, 2021. Qualtrics recruits participants through nonprobability methods including opt-in research panels, social media outreach, and specialized recruitment.^17^ Incentives may include airline miles, gift cards, or charitable donations, among other things. Survey participants were self-reported active-duty, sworn LEOs in a state or district with an ERPO law. This was originally a California-specific study but was opened to officers in other ERPO states beginning July 12, 2021, as Qualtrics was unable to recruit the target number of participants in California alone. This study was approved by the University of California, Davis Institutional Review Board. Participants read an informed consent statement and clicked on accept to indicate consent before the survey’s initiation. We followed the American Association for Public Opinion Research (AAPOR) reporting guideline for survey research.^18^

### Measures

Nearly all survey questions were close-ended and, when appropriate, response options were presented with Likert scales. After screening for eligibility, participants were asked to report their age, educational level, gender, and races and ethnicity. These demographic features were collected to facilitate our characterization of the study participants. They also were asked to indicate their political ideology and political party preference. Following this, we asked about their job: the year that they became an officer, their current job title and assignments, and characteristics of their agency.

Next, we asked participants about their familiarity with and opinions about ERPO laws. We asked whether they had ever heard of ERPOs, gun violence restraining orders, or red flag laws, and how familiar they were with these laws. We then provided a brief description of ERPO laws and asked participants to indicate their agreement with various statements about the law, such as “[ERPO]s are likely to reduce firearm violence.” We also asked which groups of people the participants thought should be permitted to petition for ERPOs.

Following this, we asked about training and experience with ERPOs. Specifically, we asked whether the participant or their department had “been directly involved” with an ERPO. We also asked whether they received training on their state’s ERPO law, how knowledgeable they felt about when and how to petition for an ERPO, and how confident they were that they could effectively serve an ERPO.

Finally, we presented the participants with 5 brief scenarios and, following each, asked about (1) the extent to which they agreed that “the police officers responding to this situation should petition the court to issue a[n ERPO]” and (2) what other action the responding officer should take. Participants could choose any combination of the following actions: make an arrest, seek a domestic violence restraining order, seek psychiatric assessment/hospitalization (eg, 5150), and take some other action; alternatively, they could select none of these. We also solicited free-text open-ended responses allowing participants to specify what action they would take if “take some other action” was selected and to explain why they agreed or disagreed with petitioning for an ERPO. The scenarios covered a range of potential ERPO situations, including intimate partner violence (IPV), suicide, homicide, and mass shootings (eTable 1 in Supplement 1 presents the full text of all scenarios). We created 2 versions of each scenario, varying a single feature of the case in each, and randomized the version presented to participants. Differences between versions of the scenarios included gender, directionality of the threat (self vs other), risk factor history, firearm type, and race. The full text of the survey instrument is included in the eMethods in Supplement 1.

### Statistical Analysis

Our analysis of close-ended questions included standard descriptive statistics and χ^2^ tests (α = .05) to make comparisons across groups, including across randomly allocated versions of each scenario. Testing was 2-sided and unpaired. We assessed randomization by testing for differences in the demographic characteristics (age, educational level, race and ethnicity, state of residence, and political ideology) of participants randomized to different scenario versions. These analyses were done in R, version 4.2.3 (R Foundation for Statistical Computing).

To analyze the open-ended questions following each scenario, we developed qualitative codebooks through an inductive, iterative review of responses, with care given to the consideration of deviant responses.^19^ Two coders were trained on each codebook who then independently coded a sample of 30 responses.^20^ We calculated Krippendorff α in Stata, version 18 (StataCorp LLC) to determine intercoder agreement for each code.^21^ We considered codes with α ≥0.80 to have good agreement, indicating that our coding frame was sufficiently specified to support its use across coders.^22^ After reaching this threshold, responses were single coded. Most codes exceeded this threshold after the first round of coding. For items below this threshold, the coders met to reconcile disagreements before independently coding an additional 30 responses. Agreement was reached through an open discussion of coding decisions, a process that may have required adjustments to the codebook (eg, merging, redefining, or expanding codes).^23^ All codes reached α ≥0.80 after a second round of coding and were then single coded. Questions with fewer responses were fully double coded, with disagreements reconciled in the manner described above.

## Results

A total of 2039 individuals started the survey. Thirty-two declined (1.6%) to participate and 1407 were ineligible (69.0%) (ie, they did not reside in an ERPO state or were not an active-duty LEO), leaving 600 eligible participants. Of these, 235 (39.2%) did not complete the survey (their available demographic characteristics are displayed in eTable 2 in Supplement 1). Another 82 individuals (13.7%) were excluded for having markers of poor data quality (ie, completing the survey multiple times or in less than half of the median response time). The final sample size was 283.

Our sample had a median age of 40 (IQR, 34-46) years and comprised mostly cisgender men (85.2%) and non-Hispanic White officers (71.4%) (Table 1). Most participants lived in California (53.7%), followed by New York (14.5%) and Florida (10.6%). Five ERPO states were not represented in the analytic sample (Hawaii, Maryland, New Mexico, Rhode Island, and Vermont). A plurality (42.0%) of participants self-identified as politically conservative.

**Table 1.** Survey Participant Characteristics

Characteristic	Participants, No. (%) (N = 283)
Age, median (IQR), y	40 (34-46)
Highest education	
<Bachelor’s degree	107 (37.8)
Bachelor’s degree	104 (36.7)
>Bachelor’s degree	72 (25.4)
Gender	
Male	241 (85.2)
Female	40 (14.1)
Transgender man	1 (0.4)
Declined	1 (0.4)
Race and ethnicity	
Asian/Pacific Islander	5 (1.8)
Black	16 (5.7)
Hispanic	45 (15.9)
White	202 (71.4)
Multiple	3 (1.1)
Other^a^	5 (1.8)
Declined	7 (2.5)
State of residence	
California	152 (53.7)
Colorado	4 (1.4)
Connecticut	4 (1.4)
Delaware	1 (0.4)
District of Columbia	2 (0.7)
Florida	30 (10.6)
Illinois	4 (1.4)
Indiana	2 (0.7)
Massachusetts	4 (1.4)
Nevada	2 (0.7)
New Jersey	9 (3.2)
New York	41 (14.5)
Oregon	1 (0.4)
Virginia	19 (6.7)
Washington	8 (2.8)
Political ideology	
Conservative	119 (42.0)
Moderate	49 (17.3)
Liberal	83 (29.3)
Declined/unknown	32 (11.3)

^a^ Other included Native American, Middle Eastern or North African, and racial or ethnic groups not listed as a response option in the survey.

Participants had been working in law enforcement for a median of 13 (IQR, 6-20) years (Table 2). A total of 59.4% worked as officers or deputies, 24.4% worked in positions of leadership (ie, supervisory roles), and 16.3% worked in other roles. At the time of the survey, 64.0% of participants were assigned to patrol/enforcement, 29.7% were assigned to specialized units (eg, special weapons and tactical teams), and 19.8% were engaged in community policing. A total of 10.2% worked in training and 10.6% had other assignments. Participants worked for agencies of varying sizes and urbanicity.

**Table 2.** Law Enforcement Employment Details

Variable	Participants, No. (%) (n = 283)
Years in law enforcement, median (IQR)	13 (6-20)
Current position	
Officer/deputy	168 (59.4)
Leadership^a^	69 (24.4)
Other	46 (16.3)
Current assignment^b^	
Patrol/enforcement (including supervision of)	181 (64.0)
Specialized units	84 (29.7)
Community policing	56 (19.8)
Training	29 (10.2)
Other	30 (10.6)
Workplace location	
Urban	188 (66.4)
Suburban	49 (17.3)
Rural	46 (16.3)
Approximate No.of officers in agency, median (IQR)	100 (22.5-870)

^a^ These positions comprise supervisory roles including supervisory investigator, sergeant, lieutenant, captain, major, commander, deputy chief, and chief.

^b^ Categories are not mutually exclusive; 27.6% of officers indicated working in at least 2 areas.

Most participants (81.3%) were somewhat or very familiar with ERPO laws (Table 3). While 56.2% of participants received ERPO training, over three-quarters reported that they were somewhat or very knowledgeable about deciding when an ERPO is appropriate (86.2%) and about the procedures involved in petitioning for an ERPO (75.6%). Just over half (52.7%) reported that their department had been directly involved in an ERPO, and 40.6% reported direct personal involvement. When asked about who should be allowed to petition for an ERPO, most (79.2%) selected law enforcement, whereas fewer than half selected mental health professionals (47.3%), family members (43.5%), and health care professionals (41.7%) (eTable 3 in Supplement 1).

**Table 3.** ERPO Knowledge, Training, and Use

Survey question	Participants, No. (%) (n = 283)
How familiar are you with ERPOs?	
Never heard of them	40 (14.1)
Heard of them but not at all familiar	13 (4.6)
Somewhat familiar	137 (48.4)
Very familiar	93 (32.9)
Have you received training on ERPOs?	
Yes	159 (56.2)
No	114 (40.3)
I don’t know	10 (3.5)
How knowledgeable are you about deciding when an ERPO is appropriate?	
Not at all knowledgeable	39 (13.8)
Somewhat knowledgeable	160 (56.5)
Very knowledgeable	84 (29.7)
How knowledgeable are you about the procedures involved in petitioning for an ERPO?	
Not at all knowledgeable	69 (24.4)
Somewhat knowledgeable	140 (49.5)
Very knowledgeable	74 (26.1)
Has your department been directly involved in an ERPO case?	
Yes	149 (52.7)
No	95 (33.6)
I don’t know	39 (13.8)
Have you been directly involved in an ERPO case?	
Yes	115 (40.6)
No	162 (57.2)
I don’t know	6 (2.1)

Abbreviation: ERPO, extreme risk protection order.

**Figure 1.**
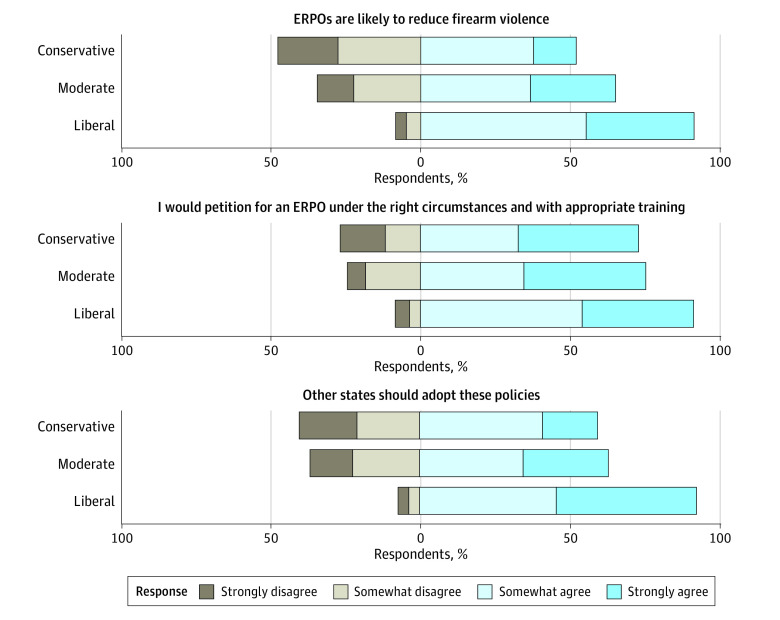
Extreme Risk Protection Order (ERPO) Opinions by Political Ideology A total of 251 of 283 participants were included; 32 participants were excluded due to unknown political ideology. For each opinion, the *P* value from a χ^2^ test for independence by political ideology was <.01.

The opinions of LEOs on ERPO laws were favorable overall but varied substantially by political ideology, with self-identified conservative participants having less favorable opinions of the law and being less willing to use it than self-identified liberal participants (Figure 1; eTable 4 in Supplement 1). For example, 91.6% of liberal participants at least somewhat agreed that “ERPOs are likely to reduce firearm violence” and 92.8% agreed that “other states should adopt these policies”; in contrast, just 52.1% of conservative participants at least somewhat agreed that ERPOs are likely to reduce firearm violence” and 59.7% agreed that “other states should adopt these policies.” Approximately one-quarter of conservative (26.9%) and moderate (24.5%) participants at least somewhat disagreed that they would “petition for an ERPO under the right circumstances and with appropriate training”; across all participants, 21.9% at least somewhat disagreed (eTable 4 in Supplement 1).

**Figure 2.**
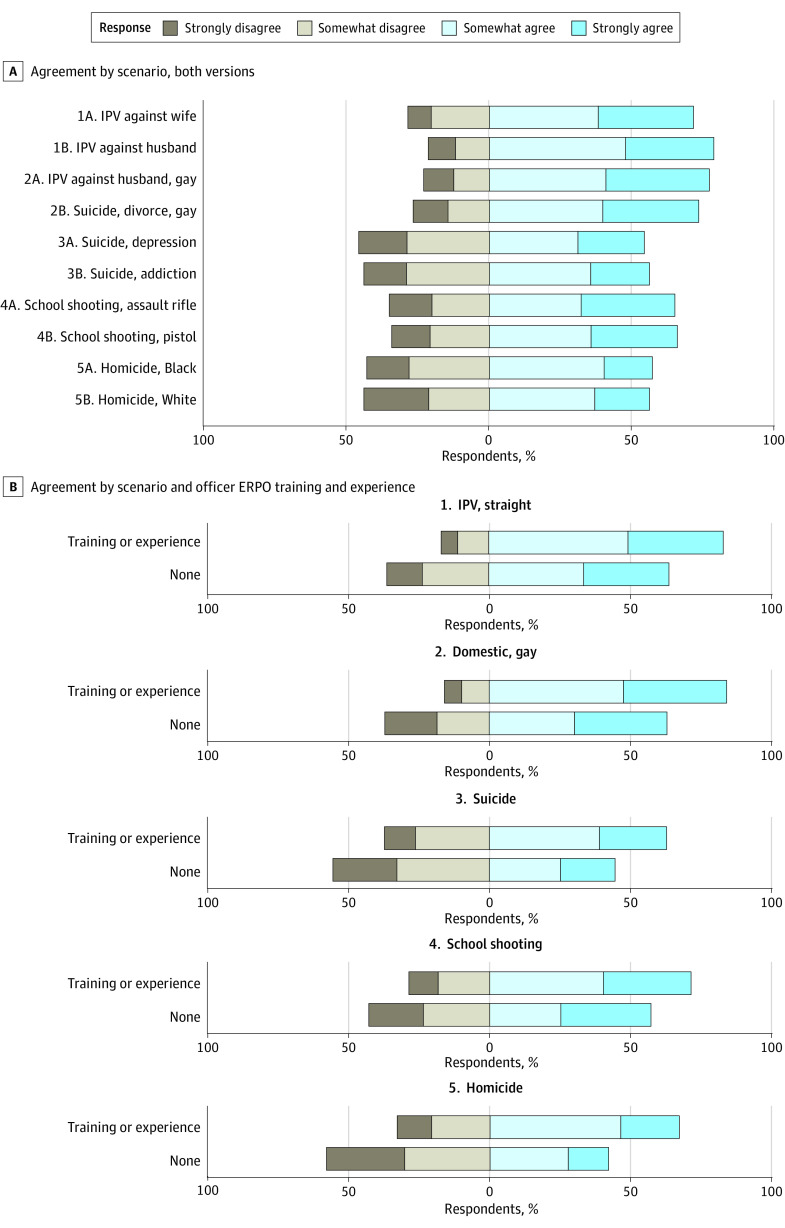
Agreement With Petitioning for an Extreme Risk Protection Order (ERPO) in Hypothetical Scenarios None of the randomized differences within scenarios were associated with statistically significant differences in agreement to petition for an ERPO. For each scenario, the *P* value from a χ^2^ test for independence by training/experience was <.05. A total of 164 participants had ERPO training or direct personal experience with an ERPO; 119 did not. IPV indicates intimate partner violence.

Participants randomized to different versions of each scenario were well balanced on demographic characteristics. Most officers at least somewhat agreed with petitioning for an ERPO across all specific scenarios (Figure 2A; eTable 5A in Supplement 1). Support for an ERPO tended to be higher in cases involving IPV (71.4%-78.6%) and lower in cases involving suicidality (54.2%-73.3%) and non-IPV interpersonal violence (56.0%-65.8%). None of the randomized differences within scenarios resulted in significant differences in agreement.

However, there were substantial differences in agreement between officers with and without ERPO training or direct personal experience with ERPOs: those with training or experience were 14 to 25 percentage points more likely to at least somewhat agree with petitioning for an ERPO across scenarios (Figure 2B; eTable 5B in Supplement 1). The differences were particularly notable in the suicide and homicide scenarios, as most of those with training or experience agreed with seeking an ERPO (suicide, 62.8%; homicide, 67.1%) and most of those without training or experience disagreed (suicide, 55.5%; homicide, 58.0%).

Participants were asked to optionally explain why they agreed or disagreed with petitioning for an ERPO after each scenario (the proportions providing explanations are presented in eTable 6A in Supplement 1). The most common reason given by those who strongly agreed with an ERPO was that there was a threat of violence (eTable 6B in Supplement 1). Among those who somewhat agreed or disagreed, the most common explanation was the need for more information. Those who strongly disagreed most commonly indicated that the ERPO was unneeded (eg, the threat was not credible).

Beyond ERPOs, we explored what other actions the participants would take in each scenario, including making an arrest, seeking a domestic violence restraining order (DVRO), and seeking a psychiatric evaluation. Most participants indicated they would take some other action in addition to or instead of an ERPO (eTable 7A in Supplement 1). In the IPV scenario in which a woman was threatened by her husband, most supported arrest (55.1%) and a DVRO (51.7%); however, the most popular other action was a DVRO (72.4%) among participants who agreed with an ERPO and arrest (50.0%) among those who disagreed (eTable 7B in Supplement 1). In the IPV scenario in which a man was threatened by his husband, most LEOs supported a DVRO (70.4%). Most supported a psychiatric evaluation in all 3 suicide scenarios (58.9%-70.2%). There was not a majority agreement about other actions that should be taken in the non-IPV interpersonal violence scenarios, and the most popular action varied by whether the participant supported petitioning for an ERPO.

## Discussion

This survey of 283 active-duty LEOs working in states with ERPO laws found that participants’ self-reported knowledge of ERPOs was high, that a little more than half had received ERPO training, and that a large minority had direct personal experience with the law. The level of ERPO knowledge, training, and use in this sample was unexpectedly high given that ERPO use is quite rare.^7^ This could suggest that ERPO use is greater than previous research reports, that participants conflated ERPOs with other protective orders, or that officers who were more familiar with ERPOs participated in the survey more often than those who were less familiar. This sample is not representative of all active-duty officers in states with ERPO laws, and our findings with regard to knowledge, training, and use likely do not generalize to that population. Nevertheless, we found 2 very interesting patterns in the data, neither of which should be influenced by this limitation, that can directly inform ERPO implementation efforts.

First, self-reported political ideology was significantly associated with officers’ opinions about and general willingness to use ERPOs, with conservative participants being more skeptical of the law and less willing to use it than liberal participants. Additionally, more than one-quarter of conservative participants disagreed that they would petition for an ERPO “under the right circumstances and with appropriate training” (compared with 8.4% of liberal participants). This may suggest an ideologically motivated refusal to use a law that, for the sake of equity, should be meaningfully available to everyone living in an ERPO state. These results bolster prior qualitative findings suggesting that personal and local politics may be a barrier to LEO uptake of ERPOs.^13^ Other research has similarly found that conservative law enforcement personnel are less likely to support firearm regulations.^24,25^

Second, we found that ERPO training or direct personal experience was associated with officers’ agreement with using ERPOs across a range of scenarios. In all scenarios, officers with training or experience were more likely to agree with using an ERPO than those without training and experience—usually by about 20 percentage points. This suggests both the necessity and value of ERPO training, as it seems to convey to officers the potential utility of ERPOs in preventing firearm violence of all kinds.

Training may also help dispel some of the misconceptions officers seem to have about when ERPOs can be used. For example, about 1 in 3 participants who strongly disagreed with using an ERPO in the school shooting scenario justified their position by noting that no illegal behavior was reported (eTable 8 in Supplement 1). Smaller proportions justified disagreeing with ERPOs in other scenarios because the threat did not involve firearms or firearms were not brandished. However, ERPO laws require neither that the respondent commit a crime nor that a firearm is used or owned (in fact, preventing the purchase of firearms is a key component of ERPOs). Similarly, a small number of officers noted concerns about ERPOs violating due process or the Second Amendment. Training could clarify that ERPO laws are widely considered and adjudicated to be constitutional.^26,27^

The results also suggest that training on how to respond to crises could be improved in general (note the variation in eTable 7A in Supplement 1) and that ERPO training could be improved, particularly with respect to clarifying the role that ERPOs can play in suicide prevention. There is evidence that ERPOs prevent firearm suicide among respondents and at the population level.^1,2,3^ However, LEOs were the least likely to agree with using an ERPO in the suicide scenarios. This reticence is also reflected in how ERPOs are being used in California, where only 15% of ERPOs in the first 3 years were issued for suicide threats alone.^4^ Extreme risk protection orders may be underused for suicide prevention in Washington and Colorado as well.^10,12^

In addition to training, procedural and structural changes may support the creation of a robust LEO ERPO implementation effort. This includes forming partnerships between police departments and city attorney’s offices, who can represent LEO petitioners; this removes the burden of filing petitions and attending court hearings from LEOs, who previously identified these things, as well as discomfort with self-representation in court, as barriers.^13^ Creating dedicated ERPO units in larger police departments can also support implementation and reduce risk of injury by having officers who are specially trained in firearm removal serve the orders. Finally, if feasible, regional ERPO teams covering many jurisdictions could promote implementation in areas served by smaller police departments that may not have the resources or demand for specialized ERPO units.

### Limitations

This study’s findings should be considered in light of its limitations. As is true of all forms of survey research, this study is potentially subject to unmeasured error. Status as an active-duty LEO was self-reported; however, given that Qualtrics uses double-opt in market research panels with screening questions to identify hard-to-reach groups, we have confidence that our participants belonged to our target population. Additionally, data are from a nonrepresentative sample and LEOs from California are overrepresented; findings may not generalize to the broader population of LEOs working in states with an ERPO law. Future studies using representative samples are warranted. Furthermore, our findings suggest possible sampling bias, potentially with LEOs more familiar with ERPOs disproportionately opting into the survey compared with those less familiar. This impacts the generalizability of the total proportions reporting knowledge of, training on, and experience with the law but is unlikely to affect our main findings. Finally, we are lacking information about the quality and extent of participants’ ERPO training. Identifying heterogeneity in training, as well as best practices, are areas ripe for research.

## Conclusions

Extreme risk protection order laws may be effective at preventing firearm violence but only to the extent that they are implemented well. Findings from this study provide insight into how those primarily responsible for implementation (ie, LEOs) perceive the law generally and in specific circumstances. These findings also reveal the importance of ERPO training or experience in LEOs’ perceptions of the law’s utility across a range of scenarios and suggest how training could be improved. As more states begin to adopt and implement these laws, additional guidance for LEO implementation will be essential.

## Notes

Supplement 1. **eTable 1.** Scenario Details **eTable 2.** Demographic Characteristics by Completion of Survey **eTable 3.** Opinions on ERPO Petitioners **eTable 4.** ERPO Opinions Overall and by Political Ideology **eTable 5.** Agreement with Petitioning for an ERPOs in Hypothetical Scenarios **eTable 6.** Reasons For and Against Petitioning for an ERPO by Scenario **eTable 7.** Suggested Further Actions **eTable 8.** Indications of Misconceptions About ERPOs Laws in Qualitative Explanations of Why/Why Not an ERPO Should Be Used in Specific Case Scenarios **eMethods.** Survey Instrument

Supplement 2.
Data Sharing Statement
