# Supplementary material for: Law Enforcement Officer Knowledge of, Attitudes Toward, and Willingness to Use Extreme Risk Protection Orders
Source: JAMA Netw Open. 2023 Oct 19;6(10):e2338455. doi: 10.1001/jamanetworkopen.2023.38455 (PMC10587793; doi:10.1001/jamanetworkopen.2023.38455)
Supplement: Supplement 1. — eTable 1. Scenario Details eTable 2. Demographic Characteristics by Completion of Survey eTable 3. Opinions on ERPO Petitioners eTable 4. ERPO Opinions Overall and by Political Ideology eTable 5. Agreement with Petitioning for an ERPOs in Hypothetical Scenarios eTable 6. Reasons For and Against Petitioning for an ERPO by Scenario eTable 7. Suggested Further Actions eTable 8. Indications of Misconceptions About ERPOs Laws in Qualitative Explanations of Why/Why Not an ERPO Should Be Used in Specific Case Scenarios eMethods. Survey Instrument [file jamanetwopen-e2338455-s001.pdf]

Supplementary Online Content

Pear VA, De Biasi A, Charbonneau A. Law enforcement officer knowledge of, attitudes toward, and willingness to use extreme risk protection orders. *JAMA Netw Open*. 2023;6(10):e2338455. doi:10.1001/jamanetworkopen.2023.38455

eTable 1. Scenario Details

eTable 2. Demographic Characteristics by Completion of Survey

eTable 3. Opinions on ERPO Petitioners

eTable 4. ERPO Opinions Overall and by Political Ideology

eTable 5. Agreement with Petitioning for an ERPOs in Hypothetical Scenarios

eTable 6. Reasons For and Against Petitioning for an ERPO by Scenario

eTable 7. Suggested Further Actions

eTable 8. Indications of Misconceptions About ERPOs Laws in Qualitative Explanations of Why/Why Not an ERPO Should Be Used in Specific Case Scenarios

eMethods. Survey Instrument

This supplementary material has been provided by the authors to give readers additional information about their work.

© 2023 Pear VA et al. *JAMA Network Open.*

eTable 1. Scenario Details

Scenario No. and Abbreviated Description	Randomized Feature	Scenario Text
1A. IPV against wife	Gender	A couple is going through a divorce and the **husband** threatens to kill his **wife** during an argument. The wife reports the threat to the police and says that her husband owns several firearms. The police have been called to the house for domestic violence in the past year.
1B. IPV against husband		A couple is going through a divorce and the **wife** threatens to kill her **husband** during an argument. The husband reports the threat to the police and says that his wife owns several firearms. The police have been called to the house for domestic violence in the past year.
2A. IPV against husband, gay	Target of threat	A gay couple is going through a divorce and one of the men threatens to **kill the other** during an argument. The threatened man reports the threat to the police and says that his husband owns several firearms. The police have been called to the house for **domestic violence** in the past year.
2B. Suicide, Divorce, gay		A gay couple is going through a divorce and one of the men threatens to **kill himself** during an argument. The other man reports the threat to the police and says that his husband owns several firearms. The police have been called to the house for **suicidal threats** in the past year.
3A. Suicide, depression	Risk factor for suicide	At his father's funeral, a person with a history of **depression** tells his sister that he'd be better off dead. He just inherited his father's firearms. His sister reports the situation to the police.
3B. Suicide, addiction		At his father's funeral, a person with a history of **addiction** tells his sister that he'd be better off dead. He just inherited his father's firearms. His sister reports the situation to the police.
4A. School shooting, assault rifle	Firearm type	Shortly after sharing a post on Facebook praising school shooters, an 18-year old high school student posts a video of himself shooting an **assault rifle (e.g., an AR or AK type rifle)** at a shooting range. The student has been suspended from school several times.
4B. School shooting, pistol		Shortly after sharing a post on Facebook praising school shooters, an 18-year old high school student posts a video of himself shooting a **pistol** at a shooting range. The student has been suspended from school several times.
5A. Homicide, Black	Race	During an altercation at a party, a 23-year-old **Black** man threatens to shoot another young man in retaliation for a previous conflict. An anonymous partygoer reported the threat and believes the man owns firearms.
5B. Homicide, white		During an altercation at a party, a 23-year-old **white** man threatens to shoot another young man in retaliation for a previous conflict. An anonymous partygoer reported the threat and believes the man owns firearms.

© 2023 Pear VA et al. *JAMA Network Open.*

eTable 2. Demographic Characteristics by Completion of Survey

	Ended Survey Early N (%), (n=235)	Completed Survey (Analytic Dataset) N (%), (n=283)	P-value[a]
**Age** (med, IQR)	42 (35, 47)	40 (34, 46)	0.2
Unknown	52		
**Highest Education**			<0.01
< Bachelor's degree	104 (57.1)	107 (37.8)	
Bachelor's degree	59 (32.4)	104 (36.7)	
> Bachelor's degree	19 (10.4)	72 (25.4)	
Unknown	53		
**Gender**			0.09
Male	146 (81.6)	241 (85.2)	
Female	28 (15.6)	40 (14.1)	
Transgender Man	0 (0.0)	1 (0.4)	
Decline	5 (2.8)	1 (0.4)	
Unknown	56		
**Race/Ethnicity**			0.03
Asian/Pacific Islander	6 (3.3)	5 (1.8)	
Black	4 (2.2)	16 (5.7)	
Hispanic	31 (16.9)	45 (15.9)	
White	121 (66.1)	202 (71.4)	
Multiple	7 (3.8)	3 (1.1)	
Other	2 (1.1)	5 (1.8)	
Decline	12 (6.6)	7 (2.5)	
Unknown	52		
**State of Residence**			0.21
California	121 (51.5)	152 (53.7)	
Colorado	0 (0)	4 (1.4)	
Connecticut	0 (0)	4 (1.4)	
Delaware	1 (0.4)	1 (0.4)	
District of Columbia	1 (0.4)	2 (0.7)	
Florida	31 (13.2)	30 (10.6)	
Illinois	4 (1.7)	4 (1.4)	
Indiana	2 (0.9)	2 (0.7)	
Maryland	3 (1.3)	0 (0)	
Massachusetts	5 (2.1)	4 (1.4)	
Nevada	1 (0.4)	2 (0.7)	
New Jersey	10 (4.3)	9 (3.2)	
New York	44 (18.7)	41 (14.5)	
Oregon	1 (0.4)	1 (0.4)	
Vermont	1 (0.4)	0 (0)	
Virginia	8 (3.4)	19 (6.7)	
Washington	2 (0.9)	8 (2.8)	

© 2023 Pear VA et al. *JAMA Network Open.*

**Political Ideology**			<0.01
Conservative	88 (56.8)	119 (45.4)	
Moderate	42 (27.1)	49 (18.7)	
Liberal	9 (5.8)	83 (31.7)	
Decline	16 (10.3)	11 (4.2)	
Unknown	80	21	

a. P-values for categorical variables are from chi-square tests or Fisher's exact tests (when the expected cell size is <5). P-values for continuous variables are from Wilcoxon rank sum tests.

© 2023 Pear VA et al. *JAMA Network Open.*

eTable 3. Opinions on ERPO Petitioners

**Who should be able to petition for an ERPO?**	N (%) Participants (n=283)
Law enforcement	224 (79.2)
Mental health professional	134 (47.3)
Family members	123 (43.5)
Health care professional	118 (41.7)
Parents or legal guardians	82 (29.0)
People who are living together	57 (20.1)
People who have a child in common	55 (19.4)
Current or former dating partner	54 (19.1)
School administrator or teacher	46 (16.3)
Employer or co-worker	41 (14.5)
None of these	17 (6.0)
Unsure	6 (2.1)
Other	3 (1.1)

© 2023 Pear VA et al. *JAMA Network Open.*

eTable 4. ERPO Opinions Overall and by Political Ideology

		Self-Identified Political Ideology[a]		
	Total N (%) (n=283)	Conservative N (%) (n=119)	Moderate N (%) (n=49)	Liberal N (%) (n=83)
**"I would petition for a ERPO under the right circumstances and with appropriate training."**				
Strongly disagree	32 (11.3)	18 (15.1)	3 (6.1)	4 (4.8)
Somewhat disagree	30 (10.6)	14 (11.8)	9 (18.4)	3 (3.6)
Somewhat agree	109 (38.5)	39 (32.8)	17 (34.7)	45 (54.2)
Strongly agree	112 (39.6)	48 (40.3)	20 (40.8)	31 (37.3)
**"ERPOs are likely to reduce firearm violence."**				
Strongly disagree	44 (15.5)	24 (20.2)	6 (12.2)	3 (3.6)
Somewhat disagree	54 (19.1)	33 (27.7)	11 (22.4)	4 (4.8)
Somewhat agree	120 (42.4)	45 (37.8)	18 (36.7)	46 (55.4)
Strongly agree	65 (23.0)	17 (14.3)	14 (28.6)	30 (36.1)
**"Other states should adopt these policies."**				
Strongly disagree	43 (15.2)	23 (19.3)	7 (14.3)	3 (3.6)
Somewhat disagree	43 (15.2)	25 (21.0)	11 (22.4)	3 (3.6)
Somewhat agree	114 (40.3)	49 (41.2)	17 (34.7)	38 (45.8)
Strongly agree	83 (29.3)	22 (18.5)	14 (28.6)	39 (47.0)

a. 32 participants (11.3%) did not report their political preference. Chi-square tests show significant differences across political ideology for all three opinions (p-value for each is <0.01).

© 2023 Pear VA et al. *JAMA Network Open.*

eTable 5. Agreement with Petitioning for an ERPOs in Hypothetical Scenarios

A. **Agreement by Scenario, Both Versions**[a]

	**The officer should petition the court for a GVRO** (N %)			
**Scenario**	**Strongly Disagree**	**Somewhat Disagree**	**Somewhat Agree**	**Strongly Agree**
1A. IPV against wife (n=147)	12 (8.2)	30 (20.4)	56 (38.1)	49 (33.3)
1B. IPV against husband (n=126)	12 (9.5)	15 (11.9)	60 (47.6)	39 (31.0)
2A. IPV, gay (n=152)	16 (10.5)	19 (12.5)	62 (40.8)	55 (36.2)
2B. Suicide, gay, divorce (n=131)	16 (12.2)	19 (14.5)	52 (39.7)	44 (33.6)
3A. Suicide, depression (n=142)	24 (16.9)	41 (28.9)	44 (31.0)	33 (23.2)
3B. Suicide, addiction (n=141)	21 (14.9)	41 (29.1)	50 (35.5)	29 (20.6)
4A. School, assault rifle (n=134)	20 (14.9)	27 (20.1)	43 (32.1)	44 (32.8)
4B. School, pistol (n=149)	20 (13.4)	31 (20.8)	53 (35.6)	45 (30.2)
5A. Homicide, Black (n=142)	21 (14.8)	40 (28.2)	57 (40.1)	24 (16.9)
5B. Homicide, white (n=141)	32 (22.7)	30 (21.3)	52 (36.9)	27 (19.1)

B. **Agreement by Scenario and Officer ERPO Training and Experience**[b]

	**The officer should petition the court for a GVRO** (N %)			
**Scenario**	**Strongly Disagree**	**Somewhat Disagree**	**Somewhat Agree**	**Strongly Agree**
IPV, Straight				
Training/Exp	9 (5.8)	17 (11.0)	76 (49.4)	52 (33.8)
None	15 (12.6)	28 (23.5)	40 (33.6)	36 (30.3)
Domestic, Gay				
Training/Exp	10 (6.1)	16 (9.8)	78 (47.6)	60 (36.6)
None	22 (18.5)	22 (18.5)	36 (30.3)	39 (32.8)
Suicide				
Training/Exp	18 (11.0)	43 (26.2)	64 (39.0)	39 (23.8)
None	27 (22.7)	39 (32.8)	30 (25.2)	23 (19.3)
School shooting				
Training/Exp	17 (10.4)	30 (18.3)	66 (40.2)	51 (31.1)
None	23 (19.3)	28 (23.5)	30 (25.2)	38 (31.9)
Homicide				
Training/Exp	20 (12.2)	34 (20.7)	76 (46.3)	34 (20.7)
None	33 (27.7)	36 (30.3)	33 (27.7)	17 (14.3)

a. None of the randomized differences within scenarios were associated with statistically significant differences in agreement to petition for an ERPO.
b. For each scenario, the chi-square tests by training/experience had a p-value < 0.05. 164 participants had ERPO training or direct personal experience with an ERPO; 119 did not.

© 2023 Pear VA et al. *JAMA Network Open.*

eTable 6. Reasons For and Against Petitioning for an ERPO, by Scenario

A. Proportion of Participants Providing Free-Text Explanation

**Scenario**	***Total*** **Proportion providing explanation, N (%)**	***Agree with ERPO*** **Proportion providing explanation, N (%)**	***Disagree with ERPO*** **Proportion providing explanation, N (%)**
IPV against wife[a]	113/147 (76.9)	75/105 (71.4)	38/42 (90.5)
Domestic, gay	215/283 (76.0)	151/213 (70.9)	64/70 (91.4)
Suicide	191/283 (67.5)	85/156 (54.5)	106/127 (83.5)
School Shooting	177/283 (62.5)	104/185 (56.2)	73/98 (74.5)
Homicide	203/283 (71.7)	95/160 (59.4)	108/123 (87.8)

B. Most Popular Reasons For and Against Petitioning for an ERPO, by Scenario

	**The officer should petition the court for an ERPO** (N %)			
**Scenario**	**Strongly Agree**	**Somewhat Agree**	**Somewhat Disagree**	**Strongly disagree**
IPV against wife[a]	Access to firearms 12/49 (24.5) & Safety/DV History 12/49 (24.5)	Additional info needed 10/56 (17.9)	Firearms would be removed for a different reason 11/30 (36.7)	Validity of allegations/threat 3/12 (25.0)
Same-sex domestic disturbance	Threat to self/others 23/99 (23.2)	Additional info needed 13/114 (11.4)	Other protective order preferred 6/38 (15.8)	Seek psych eval/ protective order 6/32 (18.8) & Firearms would be removed for a different reason 6/32 (18.8)
Suicide	Threat to self 20/62 (32.3)	Additional info needed 16/94 (17.0)	Not a credible threat 38/82 (46.3)	Not a credible threat 19/45 (42.2)
School shooting	Potential for danger 36/89 (40.4)	Further investigation 18/96 (18.8)	Further investigation 20/58 (34.5)	No illegal behavior 13/40 (32.5)
Homicide	Made (illegal) threat 12/51 (23.5)	Additional info needed 28/109 (25.7)	Additional info needed 35/70 (50.0)	Additional info needed 19/53 (35.8)

a. Results for IPV against husband (n=126) not included due to an error in the survey flow.

© 2023 Pear VA et al. *JAMA Network Open.*

eTable 7. Suggested Further Actions

A. By Scenario

	**Further Action** (N %)				
**Scenario**	**Arrest**	**DVRO**[b]	**Psych Eval**	**Other**	**Nothing Else**
IPV against wife[a] (n=147)	81 (55.1)	76 (51.7)	0 (0.0)	25 (17.0)	1 (0.7)
IPV, gay (n=152)	71 (46.7)	107 (70.4)	35 (23.0)	20 (13.2)	5 (3.3)
Suicide, divorce, gay (n=131)	32 (24.4)	36 (27.5)	92 (70.2)	13 (9.9)	1 (0.8)
Suicide, depression (n=142)	22 (15.5)	21 (14.8)	86 (60.6)	31 (21.8)	5 (3.5)
Suicide, addiction (n=141)	19 (13.5)	23 (16.3)	83 (58.9)	23 (16.3)	15 (10.6)
School, assault rifle (n=134)	26 (19.4)	18 (13.4)	64 (47.8)	40 (29.9)	16 (11.9)
School, pistol (n=149)	33 (22.1)	32 (21.5)	60 (40.3)	47 (31.5)	22 (14.8)
Homicide, Black (n=142)	64 (45.1)	23 (16.2)	19 (13.4)	45 (31.7)	21 (14.8)
Homicide, white (n=141)	65 (46.1)	34 (24.1)	22 (15.6)	40 (28.4)	15 (10.6)

B. Most Popular Further Action by Scenario and ERPO Endorsement

	**Most Popular Further Action** (N %)			
**Scenario**	**In addition to ERPO**		**Instead of ERPO**	
IPV against wife[a]	DVRO	76/105 (72.4)	Arrest	21/42 (50.0)
IPV, gay	DVRO	84/117 (71.8)	DVRO	23/35 (65.7)
Suicide, divorce, gay	Psych Eval	65/96 (67.7)	Psych Eval	27/35 (77.1)
Suicide, depression	Psych Eval	49/77 (63.6)	Psych Eval	37/65 (56.9)
Suicide, addiction	Psych Eval	47/79 (59.5)	Psych Eval	36/62 (58.1)
School, assault rifle	Psych Eval	46/87 (52.9)	Other Action[c]	20/47 (42.6)
School, pistol	Psych Eval	45/98 (45.9)	Other Action[c]	23/51 (45.1)
Homicide, Black	Arrest	52/81 (64.2)	Other Action[c]	26/61 (42.6)
Homicide, white	Arrest	43/79 (54.4)	Other Action[c]	24/62 (38.7)

a. Results for IPV against husband (n=126) not shown due to an error in the survey flow.
b. DVRO = domestic violence restraining order
c. The most popular other action was further investigation/interviewing involved parties.

© 2023 Pear VA et al. *JAMA Network Open.*

eTable 8. Indications of Misconceptions About ERPOs Laws in Qualitative Explanations of Why/Why Not an ERPO Should Be Used in Specific Case Scenarios

**Characteristic**	**Overall** (n=283) N (%)	**Strongly agree** N (%)	**Somewhat agree** N (%)	**Somewhat disagree** N (%)	**Strongly disagree** N (%)
Scenario 1: IPV against wife[a]		N=49	N=56	N=30	N=12
Weapon not brandished	7 (4.8)	0 (0)	1 (1.8)	4 (13.3)	2 (16.7)
No violence/crime	1 (0.7)	0 (0)	0 (0)	0 (0)	1 (8.3)
2nd Amendment/ constitutional rights	6 (4.1)	0 (0)	0 (0)	5 (16.7)	1 (8.3)
Scenario 2: Domestic disturbance		N=99	N=114	N=38	N=32
Threat did not involve a firearm	6 (2.1)	0 (0)	0 (0)	3 (7.9)	3 (9.4)
2nd Amendment/ constitutional rights	7 (2.5)	1 (1.0)	1 (0.9)	3 (7.9)	2 (6.2)
Scenario 3: Suicide		N=62	N=94	N=82	N=45
No crime	3 (1.1)	0 (0)	0 (0)	1 (1.2)	2 (4.4)
2nd Amendment/ constitutional rights	9 (3.2)	0 (0)	0 (0)	4 (4.9)	5 (11.1)
Scenario 4: School shooting		N=89	N=96	N=58	N=40
No crime	27 (9.5)	0 (0)	1 (1.0)	13 (22.4)	13 (32.5)
Scenario 5: Homicide		N=51	N=109	N=70	N=53
Weapon not brandished	4 (1.4)	0 (0)	0 (0)	3 (4.3)	1 (1.9)
No crime/unclear if crime was committed	7 (2.5)	0 (0)	2 (1.8)	3 (4.3)	2 (3.8)
2nd Amendment/ constitutional rights	10 (3.5)	0 (0)	0 (0)	3 (4.3)	7 (13.2)

a. Results for IPV against husband (n=126) not included due to an error in the survey flow.

© 2023 Pear VA et al. *JAMA Network Open.*

eMethods: Survey Instrument

Start of Block: Screening

Q1.1 In which state do you currently reside?

▼ Alabama (1) ... I do not reside in the United States (53)

Q1.2 Are you currently a sworn law enforcement officer working in ${Q1.1/ChoiceGroup/SelectedChoices}? (If you are retired or in the academy, please select "No")

- Yes (1)
- No (2)

End of Block: Screening

Start of Block: Demographics and professional experience

Q2.1 The first few questions are about you and your background.

Q2.2 I am ___ years old.

Q2.3 Please indicate the highest level of education that you have completed (select one):

- Less than high school diploma (1)
- High school diploma or GED (2)
- Some college (3)
- Associate's degree (4)
- Bachelor's degree (5)
- Master's degree (6)
- Professional degree (7)
- Doctorate (8)

Q2.4 I identify as (choose one):

- Male (1)
- Female (2)
- Transgender man (3)
- Transgender woman (4)
- Non-binary (5)
- An identity not listed above (6)
- Decline to state (7)

Q2.5 Do you identify as Hispanic and/or Latino?

- Yes (1)
- No (2)
- Decline to state (3)

Q2.6 I identify as (choose all that apply):

- ▢ White (1)

© 2023 Pear VA et al. *JAMA Network Open.*

- ☐ Black or African American (2)
- ☐ Native American (3)
- ☐ East Asian (4)
- ☐ Southeast Asian (5)
- ☐ South Asian (6)
- ☐ Pacific Islander or Native Hawaiian (7)
- ☐ Middle Eastern or North African (8)
- ☐ A racial/ethnic group not listed above: (9)
- ☐ ⊗Decline to state (10)

Q2.7 In general, do you think of yourself as...

- ○ Extremely liberal (1)
- ○ Liberal (2)
- ○ Slightly liberal (3)
- ○ Moderate, middle of the road (4)
- ○ Slightly conservative (5)
- ○ Conservative (6)
- ○ Extremely conservative (7)
- ○ Decline to state (8)

Q2.8 Do you have a political party preference?

- ○ The Democratic Party (1)
- ○ The Republican Party (2)
- ○ The Green Party (3)
- ○ The Libertarian Party (4)
- ○ Other (5) ______________
- ○ No political party preference (6)
- ○ Decline to state (7)

Q2.9 I was sworn in as a police officer in _____ (year).

Q2.10 My current title is (select one):

- ○ Police Officer (1)
- ○ Detective (2)
- ○ Sergeant (3)
- ○ Lieutenant (4)
- ○ Captain (5)
- ○ Commander (6)

© 2023 Pear VA et al. *JAMA Network Open.*

- ○ Deputy Chief (7)
- ○ Other: (8) ________________

Q2.11 My current assignment is (check all that apply currently):

- ▢ Vehicle patrol (1)
- ▢ Foot patrol (2)
- ▢ Bike patrol (3)
- ▢ Mounted patrol (4)
- ▢ Motorcycle patrol (5)
- ▢ School patrol or Youth resources (6)
- ▢ SWAT (7)
- ▢ K9 (8)
- ▢ Narcotics (9)
- ▢ Special Investigations (10)
- ▢ Community policing (11)
- ▢ Training (12)
- ▢ Other (13) ________________

Q2.12 I typically work in an area that is

- ○ Urban (1)
- ○ Suburban (2)
- ○ Rural (3)

Display This Question:

If 50 States, D.C. and Puerto Rico = California

Q2.13 Which county in California do you work in?
County (1)

▼ Alameda County (1) ... Yuba County (58)

Q2.14 Approximately how many sworn officers serve in your agency?

____

End of Block: Demographics and professional experience

Start of Block: GVRO Familiarity

Q3.1 The next few questions pertain to Extreme Risk Protection Orders (ERPOs), which are also known as Gun Violence Restraining Orders (GVROs), or “red flag” laws.

© 2023 Pear VA et al. *JAMA Network Open.*

Q3.2 Have you heard of ERPOs, GVROs, or red flag laws prior to taking this survey?

- ○ Yes (1)
- ○ No (2)

Display This Question:

If Have you heard of ERPOs, GVROs, or red flag laws prior to taking this survey? = Yes

Q3.3 How familiar are you with ERPOs, GVROs, or red flag laws?

- ○ Not at all familiar (1)
- ○ Somewhat familiar (2)
- ○ Very familiar (3)

End of Block: GVRO Familiarity

Start of Block: GVRO opinions

Q4.1 An ERPO, GVRO, or red flag law generally allows family members, household members, and the police to ask a judge to issue an order to temporarily remove guns and prevent the purchase of new guns by individuals who pose a significant danger of harming themselves or others and for whom alternative measures, such as arrest or psychiatric hospitalization, are inappropriate or have been ineffective. The order is temporary, usually lasting between three weeks to one year. From here on, we'll refer to all of these types of policies as "GVRO laws," which reflects the terminology used in California law.

Q4.2 For each statement, please indicate the extent to which you agree or disagree.

	Strongly disagree (1)	Somewhat disagree (2)	Somewhat agree (3)	Strongly agree (4)
I would be willing to petition the court for a GVRO under the right circumstances and with appropriate training on the process. (1)	○	○	○	○
GVROs are likely to reduce firearm violence. (2)	○	○	○	○
Other states should adopt these policies. (3)	○	○	○	○

Q4.3 Why do you think other states should or should not adopt GVRO laws?

____

© 2023 Pear VA et al. *JAMA Network Open.*

Q4.
Who do you think should be allowed to request a GVRO from a judge? (Select all that apply)

- ☐ Family members (1)
- ☐ Current or former dating partner (2)
- ☐ People who have a child in common (3)
- ☐ People who are living together (4)
- ☐ Parents or legal guardians (5)
- ☐ Law enforcement (6)
- ☐ Health care professional (7)
- ☐ Mental health professional (8)
- ☐ School administrator or teacher (9)
- ☐ Employer or co-worker (10)
- ☐ ⊗None of these (11)
- ☐ Other (please specify): (12) __________________
- ☐ ⊗Unsure (13)

End of Block: GVRO opinions

Start of Block: GVRO use and training

Q5.1 Has your department been directly involved in GVRO cases?

- ○ Yes (1)
- ○ No (2)
- ○ I don't know (3)

Q5.2 Have you noticed a change in GVRO use since the coronavirus outbreak began?

- ○ Yes, increased use (1)
- ○ Yes, decreased use (2)
- ○ No change (3)
- ○ I don't know (4)

Q5.3 Have you received training on GVROs?

- ○ Yes (1)
- ○ No (2)
- ○ I don't know (3)

© 2023 Pear VA et al. *JAMA Network Open.*

Q5.4 How knowledgeable do you feel regarding the following topics:

	Not at all knowledgeable (1)	Somewhat knowledgeable (2)	Very knowledgeable (3)
Deciding when a GVRO is appropriate (1)	○	○	○
The procedures involved in petitioning for a GVRO (2)	○	○	○

Q5.5 If your superior directed you to serve a GVRO how confident are you that you would be able to effectively do so?

- ○ Not at all confident (1)
- ○ Somewhat confident (2)
- ○ Very confident (3)

Q5.6 Have you ever been directly involved in a GVRO case?

- ○ Yes (1)
- ○ No (2)
- ○ I don't know (3)

Display This Question:

If Have you ever been directly involved in a GVRO case? = Yes

Q5.7 Approximately how many GVRO cases have you been involved in?

- ○ 1-5 (1)
- ○ 6-10 (2)
- ○ 11-20 (3)
- ○ 21-50 (4)
- ○ Over 50 (5)

End of Block: GVRO use and training

Start of Block: Scenario

Q7.1 Now we'll present brief scenarios and ask for your opinion on whether the police should pursue an GVRO. We're interested in your initial reaction to the limited amount of information provided on each situation.

End of Block: Scenario

Start of Block: Scenario 1A

© 2023 Pear VA et al. *JAMA Network Open.*

Q8.1 Scenario 1: A couple is going through a divorce and the husband threatens to kill his wife during an argument. The wife reports the threat to the police and says that her husband owns several firearms. The police have been called to the house for domestic violence in the past year.

Q8.2 Please indicate the extent to which you agree or disagree with the following:

	Strongly disagree (1)	Somewhat disagree (2)	Somewhat agree (3)	Strongly agree (6)
The police officers responding to this situation should petition the court to issue a GVRO. (1)	○	○	○	○

© 2023 Pear VA et al. *JAMA Network Open.*

Display These Two Questions:

If Please indicate the extent to which you agree or disagree with the following: = The police officers responding to this situation should petition the court to issue a GVRO. [Strongly agree]

Or Please indicate the extent to which you agree or disagree with the following: = The police officers responding to this situation should petition the court to issue a GVRO. [Somewhat agree]

Q8.4 Why do you ${Q8.2/ChoiceGroup/SelectedAnswers} that the police officer should petition for a GVRO?

____

Q8.6 In addition, the responding officers should… (check all that apply)

- ▢ Make an arrest (1)
- ▢ Seek a domestic violence restraining order (2)
- ▢ Seek psychiatric assessment/hospitalization (3)
- ▢ Take some other action (please specify): (4) ________________
- ▢ ⊗None of these (5)

Display These Two Questions:

If Please indicate the extent to which you agree or disagree with the following: = The police officers responding to this situation should petition the court to issue a GVRO. [Somewhat disagree]

Or Please indicate the extent to which you agree or disagree with the following: = The police officers responding to this situation should petition the court to issue a GVRO. [Strongly disagree]

Q8.8 Why do you ${Q8.2/ChoiceGroup/SelectedAnswers} that the police officer should petition for a GVRO?

____

Q8.10 Instead, the responding officers should… (check all that apply)

- ▢ Make an arrest (1)
- ▢ Seek a domestic violence restraining order (2)
- ▢ Seek psychiatric assessment/hospitalization (3)
- ▢ Take some other action (please specify): (4) __________________
- ▢ ⊗None of these (5)

Q8.12 Do you have any additional thoughts about this situation?

____

End of Block: Scenario 1A

Start of Block: Scenario 1B

© 2023 Pear VA et al. *JAMA Network Open.*

Q9.1 Scenario 1: A couple is going through a divorce and the wife threatens to kill her husband during an argument. The husband reports the threat to the police and says that his wife owns several firearms. The police have been called to the house for domestic violence in the past year.

Q9.2 Please indicate the extent to which you agree or disagree with the following:

	Strongly disagree (1)	Somewhat disagree (2)	Somewhat agree (3)	Strongly agree (6)
The police officers responding to this situation should petition the court to issue a GVRO. (1)	o	o	o	o

Display These Two Questions:

If Please indicate the extent to which you agree or disagree with the following: = The police officers responding to this situation should petition the court to issue a GVRO. [Somewhat agree]

Or Please indicate the extent to which you agree or disagree with the following: = The police officers responding to this situation should petition the court to issue a GVRO. [Strongly agree]

Q9.4 Why do you ${Q9.2/ChoiceGroup/SelectedAnswers} that the police officer should petition for a GVRO?

____

Q9.6 In addition, the responding officers should… (check all that apply)

▢ Make an arrest (1)

▢ Seek a domestic violence restraining order (2)

▢ Seek psychiatric assessment/hospitalization (3)

▢ Take some other action (please specify): (4) __________________

▢ ⊗None of these (5)

© 2023 Pear VA et al. *JAMA Network Open.*

Display These Two Questions:

If Please indicate the extent to which you agree or disagree with the following: = The police officers responding to this situation should petition the court to issue a GVRO. [Strongly disagree]

Or Please indicate the extent to which you agree or disagree with the following: = The police officers responding to this situation should petition the court to issue a GVRO. [Somewhat disagree]

Q9.8 Why do you ${Q9.2/ChoiceGroup/SelectedAnswers} that the police officer should petition for a GVRO?

____

Q9.10 Instead, the responding officers should… (check all that apply)

- ▢ Make an arrest (1)
- ▢ Seek a domestic violence restraining order (2)
- ▢ Seek psychiatric assessment/hospitalization (3)
- ▢ Take some other action (please specify): (4) __________________
- ▢ ⊗None of these (5)

Q9.12 Do you have any additional thoughts about this situation?

____

End of Block: Scenario 1B

Start of Block: Scenario 2A

Q10.1 Scenario 2: A gay couple is going through a divorce and one of the men threatens to kill the other during an argument. The threatened man reports the threat to the police and says that his husband owns several firearms. The police have been called to the house for domestic violence in the past year.

Q10.2 Please indicate the extent to which you agree or disagree with the following:

	Strongly disagree (1)	Somewhat disagree (2)	Somewhat agree (3)	Strongly agree (6)
The police officers responding to this situation should petition the court to issue a GVRO. (1)	○	○	○	○

Display These Two Questions:

© 2023 Pear VA et al. *JAMA Network Open.*

If Please indicate the extent to which you agree or disagree with the following: = The police officers responding to this situation should petition the court to issue a GVRO. [Strongly agree]

Or Please indicate the extent to which you agree or disagree with the following: = The police officers responding to this situation should petition the court to issue a GVRO. [Somewhat agree]

Q10.4 Why do you ${Q10.2/ChoiceGroup/SelectedAnswers} that the police officer should petition for a GVRO?

____

Q10.6 In addition, the responding officers should… (check all that apply)

- ▢ Make an arrest (1)
- ▢ Seek a domestic violence restraining order (2)
- ▢ Seek psychiatric assessment/hospitalization (3)
- ▢ Take some other action (please specify): (4) ____
- ▢ ⊗None of these (5)

Display These Two Questions:

If Please indicate the extent to which you agree or disagree with the following: = The police officers responding to this situation should petition the court to issue a GVRO. [Somewhat disagree]

Or Please indicate the extent to which you agree or disagree with the following: = The police officers responding to this situation should petition the court to issue a GVRO. [Strongly disagree]

Q10.8 Why do you ${Q10.2/ChoiceGroup/SelectedAnswers} that the police officer should petition for a GVRO?

____

Q10.10 Instead, the responding officers should… (check all that apply)

- ▢ Make an arrest (1)
- ▢ Seek a domestic violence restraining order (2)
- ▢ Seek psychiatric assessment/hospitalization (3)
- ▢ Take some other action (please specify): (4) ____
- ▢ ⊗None of these (5)

Q10.12 Do you have any additional thoughts about this situation?

____

End of Block: Scenario 2A

Start of Block: Scenario 2B

© 2023 Pear VA et al. *JAMA Network Open.*

Q11.1 Scenario 2: A gay couple is going through a divorce and one of the men threatens to kill himself during an argument. The other man reports the threat to the police and says that his husband owns several firearms. The police have been called to the house for suicidal threats in the past year.

Q11.2 Please indicate the extent to which you agree or disagree with the following:

	Strongly disagree (1)	Somewhat disagree (2)	Somewhat agree (3)	Strongly agree (6)
The police officers responding to this situation should petition the court to issue a GVRO. (1)	○	○	○	○

Display These Two Questions:

If Please indicate the extent to which you agree or disagree with the following: = The police officers responding to this situation should petition the court to issue a GVRO. [Strongly agree]

Or Please indicate the extent to which you agree or disagree with the following: = The police officers responding to this situation should petition the court to issue a GVRO. [Somewhat agree]

Q11.4 Why do you ${Q11.2/ChoiceGroup/SelectedAnswers} that the police officer should petition for a GVRO?

____

Q11.6 In addition, the responding officers should… (check all that apply)

- ▢ Make an arrest (1)
- ▢ Seek a domestic violence restraining order (2)
- ▢ Seek psychiatric assessment/hospitalization (3)
- ▢ Take some other action (please specify): (4) ________________________
- ▢ ⊗None of these (5)

Display These Two Questions:

If Please indicate the extent to which you agree or disagree with the following: = The police officers responding to this situation should petition the court to issue a GVRO. [Somewhat disagree]

Or Please indicate the extent to which you agree or disagree with the following: = The police officers responding to this situation should petition the court to issue a GVRO. [Strongly disagree]

Q11.8 Why do you ${Q11.2/ChoiceGroup/SelectedAnswers} that the police officer should petition for a GVRO?

© 2023 Pear VA et al. *JAMA Network Open.*

Q11.10 Instead, the responding officers should… (check all that apply)

- ▢ Make an arrest (1)
- ▢ Seek a domestic violence restraining order (2)
- ▢ Seek psychiatric assessment/hospitalization (3)
- ▢ Take some other action (please specify): (4) ________________________
- ▢ ⊗None of these (5)

Q11.12 Do you have any additional thoughts about this situation?

____

End of Block: Scenario 2B

Start of Block: Scenario 3A

Q12.1 Scenario 3: At his father's funeral, a person with a history of depression tells his sister that he'd be better off dead. He just inherited his father's firearms. His sister reports the situation to the police.

Q12.2 Please indicate the extent to which you agree or disagree with the following:

	Strongly disagree (1)	Somewhat disagree (2)	Somewhat agree (3)	Strongly agree (6)
The police officers responding to this situation should petition the court to issue a GVRO. (1)	○	○	○	○

Display These Two Questions:

If Please indicate the extent to which you agree or disagree with the following: = The police officers responding to this situation should petition the court to issue a GVRO. [Strongly agree]

Or Please indicate the extent to which you agree or disagree with the following: = The police officers responding to this situation should petition the court to issue a GVRO. [Somewhat agree]

Q12.4 Why do you ${Q12.2/ChoiceGroup/SelectedAnswers} that the police officer should petition for a GVRO?

____

Q12.6 In addition, the responding officers should… (check all that apply)

© 2023 Pear VA et al. *JAMA Network Open.*

☐ Make an arrest (1)
☐ Seek a domestic violence restraining order (2)
☐ Seek psychiatric assessment/hospitalization (3)
☐ Take some other action (please specify): (4) ________________________
☐ ⊗None of these (5)

Display These Two Questions:

If Please indicate the extent to which you agree or disagree with the following: = The police officers responding to this situation should petition the court to issue a GVRO. [Somewhat disagree]

Or Please indicate the extent to which you agree or disagree with the following: = The police officers responding to this situation should petition the court to issue a GVRO. [Strongly disagree]

Q12.8 Why do you ${Q12.2/ChoiceGroup/SelectedAnswers} that the police officer should petition for a GVRO?

____

Q12.10 Instead, the responding officers should… (check all that apply)

☐ Make an arrest (1)
☐ Seek a domestic violence restraining order (2)
☐ Seek psychiatric assessment/hospitalization (3)
☐ Take some other action (please specify): (4) ________________________
☐ ⊗None of these (5)

Q12.12 Do you have any additional thoughts about this situation?

____

End of Block: Scenario 3A

Start of Block: Scenario 3B

Q13.1 Scenario 3: At his father’s funeral, a person with a history of addiction tells his sister that he’d be better off dead. He just inherited his father’s firearms. His sister reports the situation to the police.

Q13.2 Please indicate the extent to which you agree or disagree with the following:

	Strongly disagree (1)	Somewhat disagree (2)	Somewhat agree (3)	Strongly agree (6)
The police officers responding to this situation should petition the court to issue a GVRO. (1)	○	○	○	○

Display These Two Questions:

If Please indicate the extent to which you agree or disagree with the following: = The police officers responding to this situation should petition the court to issue a GVRO. [Strongly agree]

Or Please indicate the extent to which you agree or disagree with the following: = The police officers responding to this situation should petition the court to issue a GVRO. [Somewhat agree]

Q13.4 Why do you ${Q13.2/ChoiceGroup/SelectedAnswers} that the police officer should petition for a GVRO?

____

Q13.6 In addition, the responding officers should… (check all that apply)

- ▢ Make an arrest (1)
- ▢ Seek a domestic violence restraining order (2)
- ▢ Seek psychiatric assessment/hospitalization (3)
- ▢ Take some other action (please specify): (4) ____
- ▢ ⊗None of these (5)

Display These Two Questions:

If Please indicate the extent to which you agree or disagree with the following: = The police officers responding to this situation should petition the court to issue a GVRO. [Strongly disagree]

Or Please indicate the extent to which you agree or disagree with the following: = The police officers responding to this situation should petition the court to issue a GVRO. [Somewhat disagree]

Q13.8 Why do you ${Q13.2/ChoiceGroup/SelectedAnswers} that the police officer should petition for a GVRO?

____

Q13.10 Instead, the responding officers should… (check all that apply)

- ▢ Make an arrest (1)
- ▢ Seek a domestic violence restraining order (2)
- ▢ Seek psychiatric assessment/hospitalization (3)
- ▢ Take some other action (please specify): (4) ____
- ▢ ⊗None of these (5)

Q13.12 Do you have any additional thoughts about this situation?

____

End of Block: Scenario 3B

Start of Block: Scenario 4A

Q14.1 Scenario 4: Shortly after sharing a post on Facebook praising school shooters, an 18-year old high school student posts a video of himself shooting an assault rifle (e.g., an AR or AK type rifle) at a shooting range. The student has been suspended from school several times.

Q14.2 Please indicate the extent to which you agree or disagree with the following:

	Strongly disagree (1)	Somewhat disagree (2)	Somewhat agree (3)	Strongly agree (6)
The police officers responding to this situation should petition the court to issue a GVRO. (1)	○	○	○	○

Display These Two Questions:

If Please indicate the extent to which you agree or disagree with the following: = The police officers responding to this situation should petition the court to issue a GVRO. [Strongly agree]

Or Please indicate the extent to which you agree or disagree with the following: = The police officers responding to this situation should petition the court to issue a GVRO. [Somewhat agree]

Q14.4 Why do you ${Q14.2/ChoiceGroup/SelectedAnswers} that the police officer should petition for a GVRO?

____

Q14.6 In addition, the responding officers should… (check all that apply)

- ▢ Make an arrest (1)
- ▢ Seek a domestic violence restraining order (2)

© 2023 Pear VA et al. *JAMA Network Open.*

- ☐ Seek psychiatric assessment/hospitalization (3)
- ☐ Take some other action (please specify): (4) ____
- ☐ ⊗None of these (5)

Display These Two Questions:

If Please indicate the extent to which you agree or disagree with the following: = The police officers responding to this situation should petition the court to issue a GVRO. [Somewhat disagree]

Or Please indicate the extent to which you agree or disagree with the following: = The police officers responding to this situation should petition the court to issue a GVRO. [Strongly disagree]

Q14.8 Why do you ${Q14.2/ChoiceGroup/SelectedAnswers} that the police officer should petition for a GVRO?

____

Q14.10 Instead, the responding officers should… (check all that apply)

- ☐ Make an arrest (1)
- ☐ Seek a domestic violence restraining order (2)
- ☐ Seek psychiatric assessment/hospitalization (3)
- ☐ Take some other action (please specify): (4) ____
- ☐ ⊗None of these (5)

Q14.12 Do you have any additional thoughts about this situation?

____

End of Block: Scenario 4A

Start of Block: Scenario 4B

Q15.1 Scenario 4: Shortly after sharing a post on Facebook praising school shooters, an 18-year old high school student posts a video of himself shooting a pistol at a shooting range. The student has been suspended from school several times.

Q15.2 Please indicate the extent to which you agree or disagree with the following:

	Strongly disagree (1)	Somewhat disagree (2)	Somewhat agree (3)	Strongly agree (6)
The police officers responding to this situation should petition the court to issue a GVRO. (1)	○	○	○	○

Display These Two Questions:

If Please indicate the extent to which you agree or disagree with the following: = The police officers responding to this situation should petition the court to issue a GVRO. [Strongly agree]

Or Please indicate the extent to which you agree or disagree with the following: = The police officers responding to this situation should petition the court to issue a GVRO. [Somewhat agree]

Q15.4 Why do you ${Q15.2/ChoiceGroup/SelectedAnswers} that the police officer should petition for a GVRO?

____

Q15.6 In addition, the responding officers should… (check all that apply)

- ▢ Make an arrest (1)
- ▢ Seek a domestic violence restraining order (2)
- ▢ Seek psychiatric assessment/hospitalization (3)
- ▢ Take some other action (please specify): (4) ________________________________
- ▢ ⊗None of these (5)

Display These Two Questions:

If Please indicate the extent to which you agree or disagree with the following: = The police officers responding to this situation should petition the court to issue a GVRO. [Somewhat disagree]

Or Please indicate the extent to which you agree or disagree with the following: = The police officers responding to this situation should petition the court to issue a GVRO. [Strongly disagree]

Q15.8 Why do you ${Q15.2/ChoiceGroup/SelectedAnswers} that the police officer should petition for a GVRO?

____

Q15.10 Instead, the responding officers should… (check all that apply)

- ▢ Make an arrest (1)
- ▢ Seek a domestic violence restraining order (2)
- ▢ Seek psychiatric assessment/hospitalization (3)

☐ Take some other action (please specify): (4) ________________________________

☐ ⊗None of these (5)

Q15.12 Do you have any additional thoughts about this situation?

____

End of Block: Scenario 4B

Start of Block: Scenario 5A

Q16.1 Scenario 5: During an altercation at a party, a 23-year-old Black man threatens to shoot another young man in retaliation for a previous conflict. An anonymous partygoer reported the threat and believes the man owns firearms.

Q16.2 Please indicate the extent to which you agree or disagree with the following:

	Strongly disagree (1)	Somewhat disagree (2)	Somewhat agree (3)	Strongly agree (6)
The police officers responding to this situation should petition the court to issue a GVRO. (1)	○	○	○	○

Display These Two Questions:

If Please indicate the extent to which you agree or disagree with the following: = The police officers responding to this situation should petition the court to issue a GVRO. [Strongly agree]

Or Please indicate the extent to which you agree or disagree with the following: = The police officers responding to this situation should petition the court to issue a GVRO. [Somewhat agree]

Q16.4 Why do you ${Q16.2/ChoiceGroup/SelectedAnswers} that the police officer should petition for a GVRO?

____

Q16.6 In addition, the responding officers should… (check all that apply)

☐ Make an arrest (1)

☐ Seek a domestic violence restraining order (2)

☐ Seek psychiatric assessment/hospitalization (3)

☐ Take some other action (please specify): (4) ________________________

☐ ⊗None of these (5)

© 2023 Pear VA et al. *JAMA Network Open.*

Display These Two Questions:

If Please indicate the extent to which you agree or disagree with the following: = The police officers responding to this situation should petition the court to issue a GVRO. [Strongly disagree]

Or Please indicate the extent to which you agree or disagree with the following: = The police officers responding to this situation should petition the court to issue a GVRO. [Somewhat disagree]

Q16.8 Why do you ${Q16.2/ChoiceGroup/SelectedAnswers} that the police officer should petition for a GVRO?

____

Q16.10 Instead, the responding officers should… (check all that apply)

- ▢ Make an arrest (1)
- ▢ Seek a domestic violence restraining order (2)
- ▢ Seek psychiatric assessment/hospitalization (3)
- ▢ Take some other action (please specify): (4) ____
- ▢ ⊗None of these (5)

Q16.12 What race/ethnicity did you imagine the potential victim to be?

- ○ Non-Hispanic White (1)
- ○ Non-Hispanic Black or African American (2)
- ○ Hispanic (3)
- ○ Native American (4)
- ○ Asian or Pacific Islander (5)
- ○ Middle Eastern or North African (6)
- ○ A racial/ethnic group not listed above (7) ____
- ○ I'm not sure (8)

Q16.14 Do you have any additional thoughts about this situation?

____

End of Block: Scenario 5A

Start of Block: Scenario 5B

Q17.1 Scenario 5: During an altercation at a party, a 23-year-old white man threatens to shoot another young man in retaliation for a previous conflict. An anonymous partygoer reported the threat and believes the man owns firearms.

Q17.2 Please indicate the extent to which you agree or disagree with the following:

© 2023 Pear VA et al. *JAMA Network Open.*

	Strongly disagree (1)	Somewhat disagree (2)	Somewhat agree (3)	Strongly agree (6)
The police officers responding to this situation should petition the court to issue a GVRO. (1)	○	○	○	○

Display These Two Questions:

If Please indicate the extent to which you agree or disagree with the following: = The police officers responding to this situation should petition the court to issue a GVRO. [Strongly agree]

Or Please indicate the extent to which you agree or disagree with the following: = The police officers responding to this situation should petition the court to issue a GVRO. [Somewhat agree]

Q17.4 Why do you ${Q17.2/ChoiceGroup/SelectedAnswers} that the police officer should petition for a GVRO?

____

Q17.6 In addition, the responding officers should… (check all that apply)

- ▢ Make an arrest (1)
- ▢ Seek a domestic violence restraining order (2)
- ▢ Seek psychiatric assessment/hospitalization (3)
- ▢ Take some other action (please specify): (4) ________________________
- ▢ ⊗None of these (5)

Display These Two Questions:

If Please indicate the extent to which you agree or disagree with the following: = The police officers responding to this situation should petition the court to issue a GVRO. [Somewhat disagree]

Or Please indicate the extent to which you agree or disagree with the following: = The police officers responding to this situation should petition the court to issue a GVRO. [Strongly disagree]

Q17.8 Why do you ${Q17.2/ChoiceGroup/SelectedAnswers} that the police officer should petition for a GVRO?

____

Q17.10 Instead, the responding officers should… (check all that apply)

- ▢ Make an arrest (1)
- ▢ Seek a domestic violence restraining order (2)
- ▢ Seek psychiatric assessment/hospitalization (3)
- ▢ Take some other action (please specify): (4) ________________________

© 2023 Pear VA et al. *JAMA Network Open.*

- ☐ ⊗None of these (5)

Q17.12 What race/ethnicity did you imagine the potential victim to be?

- ○ Non-Hispanic White (1)
- ○ Non-Hispanic Black or African American (2)
- ○ Hispanic (3)
- ○ Native American (4)
- ○ Asian or Pacific Islander (5)
- ○ Middle Eastern or North African (6)
- ○ A racial/ethnic group not listed above (7) ____
- ○ I'm not sure (8)

Q17.14 Do you have any additional thoughts about this situation?

____

End of Block: Scenario 5B

© 2023 Pear VA et al. *JAMA Network Open.*
