# Supplementary material for: Law Enforcement Officer Knowledge of, Attitudes Toward, and Willingness to Use Extreme Risk Protection Orders
Source: JAMA Netw Open. 2023 Oct 19;6(10):e2338455. doi: 10.1001/jamanetworkopen.2023.38455 (PMC10587793; doi:10.1001/jamanetworkopen.2023.38455)
Supplement: Supplement 2. — Data Sharing Statement [file jamanetwopen-e2338455-s002.pdf]

Data Sharing Statement

Pear. Law Enforcement Officer Knowledge of, Attitudes Toward, and Willingness to Use Extreme Risk Protection Orders. *JAMA Netw Open*. Published October 19, 2023. doi:10.1001/jamanetworkopen.2023.38455

Data
Data available: No

Additional Information
Explanation for why data not available: Individual-level data will not be released per the terms of our IRB.
